# Clarifying the Role of Negative Emotions in the Origin and Control of Impulsive Actions

**DOI:** 10.5334/pb.502

**Published:** 2020-01-02

**Authors:** Charlotte Eben, Joël Billieux, Frederick Verbruggen

**Affiliations:** 1 Department of Experimental Psychology, Ghent University, BE; 2 Institute of Psychology, University of Lausanne, Lausanne, CH; 3 Health and Behaviour Institute, University of Luxembourg, Esch-sur-Alzette, LU

**Keywords:** impulsive action, emotion, impulsivity, action control

## Abstract

This critical review elaborates on the origin of impulsive actions and how these can be controlled. We focus in particular on the role of negative events. First, we outline how impulsive actions often originate from negative events that are (emotionally) appraised. A discrepancy between this current state and a desired goal state leads to action tendencies. The urgency of the resulting action depends on the importance of the goal and the size of the discrepancy. Second, we discuss how such impulsive actions can be regulated or controlled e.g. by biasing competition between different options, or by completely suppressing all motor output. Importantly, such control mechanisms might also depend on emotional factors. To reconcile these findings, we present a coherent theoretical framework, taking into account various cognitive, affective, and motivational mechanisms as well as contextual factors that play a crucial role in the origin and control of impulsive actions.

## Introduction

Impulsivity is a very broad and popular concept in folk psychology and many scientific (sub)disciplines, including clinical and personality psychology, psychiatry, animal research, and neurosciences. The concept generally refers to a wide range of actions or behaviors “that are poorly conceived, prematurely expressed, unduly risky, or inappropriate to the situation and that often result in undesirable outcomes” (Evenden, 1999, p. 348). Impulsivity has been associated with many psychopathological disorders and behavioral problems, including Attention Deficit Hyperactivity Disorder (ADHD), conduct disorder, substance use and abuse, behavioral addictions (such as gambling and gaming disorders), antisocial personality disorder, or bipolar disorder (Berg, Latzman, Bliwise, & Lilienfeld, 2015). Nowadays, it is widely recognized that impulsivity is a multifaceted construct and that impulsive behaviors are modulated by a wide range of cognitive, affective, and motivational processes. This critical review will focus on one facet of this construct, namely impulsive action. In the literature, impulsive action is typically associated with failures of response inhibition, or more broadly, failures in executive (i.e. voluntary) control (e.g. Bari & Robbins, 2013). However, such control accounts have little to say about how impulsive actions originate in the first place. Furthermore, recent data (including a direct replication) seem to challenge some of the core assumptions of dominant control accounts. In this critical review, we will therefore discuss how impulsive actions might originate and how these can be controlled in different ways.

## Facets of impulsivity

The construct of impulsivity generally refers either to a *trait*, which is (at least in humans) typically assessed with self-reported questionnaires, or to a *state*, which is typically assessed in laboratory tasks. Impulsivity as a trait refers to a relatively stable personality characteristic that is influenced by learning, environment, and genetic factors (Bezdjian, Baker, & Tuvblad, 2011). Several questionnaires have been developed to assess impulsivity traits. A questionnaire (and accompanying framework) that became dominant in the last years is the UPPS Impulsive Behavior Scale. Based on a range of impulsivity scales and the Big Five questionnaire (Costa Jr & McCrae, 1992), Whiteside and Lynam (2001) identified four different facets of trait impulsivity, namely (1) Urgency (defined as the tendency to commit rash or regrettable actions as a result of intense negative affect), (2) lack of Perseverance (defined as the difficulty to remain focused on cognitively demanding or boring tasks), (3) lack of Premeditation (defined as the tendency not to think and reflect on the consequences of an act before engaging in that act) and (4) Sensation seeking (defined as the tendency to look for exciting and/or new activities). Cyders and Smith (2007, 2008) further fine-tuned this scale (UPPS-P), and argued that urgency itself consists of two subcomponents, namely ‘positive’ and ‘negative’ urgency, depending on the valence of the emotional states promoting impulsive behaviors. For example, drinking too much on alcohol after you obtained a major grant could be seen as positive urgency, whereas drinking too much because your paper got rejected, might be due to negative urgency. Of all UPPS-P subscales, negative urgency has arguably received the most attention because it correlates with a wide range of behavioral problems and psychopathological symptoms (see Smith & Cyders, 2016 for a review); this association seems less pronounced for positive urgency. For example, Berg et al. (2015) showed in a meta-analysis that urgency, and negative urgency in particular, correlates with various forms of psychopathology, including problem gambling, substance abuse or mood disorders. No such strong or consistent correlations were found with other subscales or ‘subtraits’. Thus, the tendency to act rashly in response to negative emotional events seems a particularly prominent characteristic of many psychopathologies. More generally, the findings of the meta-analysis are in line with other findings showing that dysregulated behaviors are often triggered by a negative emotional event (Billieux, Gay, Rochat, & Van der Linden, 2010; Selby, Anestis, & Joiner, 2008).

Impulsivity does not only refer to traits, but also to specific states (i.e. short-term and transient changes in behavior). At a state level, often a further distinction is made between impulsive choice and impulsive action (Bari & Robbins, 2013; van Gaalen, Brueggeman, Bronius, Schoffelmeer, & Vanderschuren, 2006; van Gaalen, van Koten, Schoffelmeer, & Vanderschuren, 2006). First, a choice is considered to be impulsive when people choose a small immediate reward over a larger delayed one (but see e.g. Dalley and Robbins, 2017, for more fine-grained distinctions). Traditionally, this behavior is measured with a delay discounting task, in which participants need to choose between hypothetical immediate small monetary rewards (e.g. 10 euro now) and delayed larger ones (e.g. 15 euro in a month; Kirby & Marakovic, 1996). Second, an action is considered to be impulsive when it is executed without much forethought. Traditionally, impulsive actions are measured in response-inhibition tasks (at least in humans; see below). Generally, correlational and neuroscience research indicates that impulsive actions should be dissociated from impulsive choice (e.g. distinct neurotransmitter systems are involved in impulsive choice and impulsive actions, see Bari & Robbins, 2013 for an overview).

Here we focus on the origin and control of impulsive actions at a behavioral level. **Textbox 1** describes an example of impulsive action in daily life, and most readers can presumably think of other examples from their own past or imagine situations in which they may act impulsively. Yet, how exactly specific impulsive actions originate has been rarely studied in a systematic fashion. Work discussed above on impulsive traits suggests that negative urgency correlates with many behavioral problems. Thus, negative emotional events seem an important contributor to impulsive acts, which is why this will be a central focus of the review.

**Textbox 1: Impulsive action in daily life.**

Imagine a Monday morning. You are tired after a busy weekend and realize that you have a long day of meetings ahead of you. To make matters worse, your coffee machine does not seem to work. When you press the button, nothing happens. You try again. And again…Andagain… Eventually you snap and smash the machine. Due to a lack of impulse control, no more coffee for today (if only you had checked whether the machine was plugged in). This is a good example of an impulsive action: it is executed without much forethought (i.e. smashing the machine does not solve the main problem), it occurred in response to a negative (emotional) event(no caffeine on a Monday morning), and has a sense of urgency.

## The origin of impulsive actions

### A traditional measurement of impulsive action: response inhibition and prepotency

In the cognitive, psychiatric, and neuroscience literature, ‘impulsive action’ is typically associated with the inhibition of well-practiced and prepotent responses (Bari & Robbins, 2013; Hofmann, Friese, & Strack, 2009; Metcalfe & Mischel, 1999). This ability to inhibit responses is typically assessed in tasks such as the stop-signal task (Logan & Cowan, 1984; Verbruggen & Logan, 2008) or the go/no-go task (Donders, 1969), in which participants are instructed to execute a prepotent response on the majority of trials but cancel it when an infrequent external stop- or no-go signal is presented. Only a few studies have systematically explored how prepotency (i.e. the strong tendency to act) develops.

In a developmental response-inhibition study, Simpson et al. (2012) presented images in a Stroop-like task. In this task, images were mapped onto a specific word. For example, when children saw an image of a car, they had to say ‘book’, or when they saw an image of a dog, they had to say ‘cat’. Simpson et al. found that response-set overlap (e.g. there were images of books and cars, and children had to say ‘book’ to car images, and ‘car’ to book images) resulted in longer response latencies and higher error rates, compared to a baseline condition without overlap, or a condition with semantic overlap (e.g. there were images of dogs and hands, but children had to say ‘cat’ to dog images and ‘foot’ to hand images). The latter conditions did not differ. This led the authors to conclude that responses became prepotent when children had the intention to produce them at some point (for related findings in younger and older children, see e.g. Verbruggen, McLaren, Pereg, & Meiran, 2018).

Methodological response-inhibition reviews (e.g. Verbruggen et al., 2019) indicate that prepotency can also be induced via task instructions (‘respond as quickly as possible’) and certain design choices (e.g. presenting stop signals only on a minority of the trials, using a strict response deadline,…). For example, in stop-signal tasks (one of the most popular paradigms to study response inhibition), subjects are typically instructed to respond as quickly as possible and not to wait for the infrequent stop signal; this is required to obtain reliable estimates of the covert latency of the stop process (see e.g. Verbruggen, Chambers, & Logan, 2013). When a response is prepotent, it becomes more difficult to inhibit. This link between prepotency and inhibition is formalized by the independent race model of Logan and colleagues (Logan & Cowan, 1984; Logan, Van Zandt, Verbruggen, & Wagenmakers, 2014).

In sum, research on response inhibition suggests that prepotency can be created via task instructions and certain task characteristics. However, most of this response-inhibition research used neutral stimuli (below we discuss a few exceptions) and external signals, so questions can be raised about the validity of the tasks. In particular, the personality and clinical research discussed above indicates that emotional or motivationally salient events are an important trigger of impulsive behaviors. Thus, standard response-inhibition tasks may not reveal much about the origins of impulsive action in non-laboratory settings (although it should be noted that some studies did find correlations between the urgency subscale of the UPPS and behavioral performance in response-inhibition tasks; Cyders & Coskunpinar, 2012; Gay, Rochat, Billieux, d’Acremont, & Van der Linden, 2008; Wilbertz et al., 2014).

### The emotional origin of impulsive action

In this review we will refer to ‘emotions’ in sense that Frijda (2010) defined them. One of the defining features of emotions according to Frijda (2010), is that they lead to actions. This happens in different steps. First, events would be ‘appraised’ by the individual. Appraisal in this case means that the individual makes a fast judgement of the situation based on previous experiences and representations; and as a result, the event becomes ‘emotional’ and thus meaningful to the individual. Second, the appraised emotional events form a state of ‘action readiness’, which is a state to establish, change or sustain the individual’s relation to the event. Such states *might* induce an action in order to achieve one of two aims, namely approaching a desired state or avoiding an unwanted one. Third, the imbalance between the current and the desired state will elicit arousal, producing an autonomic response (such as changes in heart rate, ‘frowning response’ over facial muscles, skin conductance or pupillary response). The autonomic responses are displayed in anticipation and preparation of the possible actions, and might eventually trigger them to change the current goal state. In other words, in Frijda’s framework, ‘degree of arousal’ corresponds to the mobilization of resources and leads to a ‘sense of urgency’. This also implies that emotional events have two different dimensions: On the one hand the valence (positive or negative) and on the other hand the arousal they evoke (activation vs. deactivation). The idea that emotional events can be accounted for by two comparable orthogonal dimensions can be found in several other theories as well (Inzlicht, Bartholow, & Hirsh, 2015; Russell & Barrett, 1999; Saunders & Inzlicht, 2015).

Frijda’s (2010) main ideas received support from several lines of evidence. For example, facial electromyography (EMG) has been used to investigate how an event becomes appraised. In many studies, activation of the corrugator supercilii (muscle involved in frowning) has been related to the processing of negative stimuli and emotions, whereas activation of the zygomatic muscle (muscle involved in laughing and smiling) has been mostly related to the processing of positive stimuli and emotions (e.g. Mauss & Robinson, 2009). These strong associations have been used to determine how certain events are emotionally appraised (although one should of course be aware of potential reverse inference problems; see e.g. Poldrack, 2006, for a related discussion). For example, Wu, van Dijk and Clark (2015) measured in a gambling task self-perceived luck, betting behavior, and facial muscle reactivity over the zygomaticus and the corrugator. Activity of the zygomaticus (the ‘smiling muscle’) did not systematically correlate with specific events in their task. However, activity of the corrugator (the ‘frowning muscle’) increased with losses, which led the authors to conclude that losses are appraised as (intense) emotional events. As discussed in more detail below, errors in simple choice-discrimination tasks have a similar effect on corrugator activity (Elkins-Brown, Saunders, & Inzlicht, 2016; Lindström, Mattsson-Mårn, Golkar, & Olsson, 2013).

The idea that emotional stimuli or events lead to action readiness received support from transcranial magnetic stimulation (TMS) studies. When a brief TMS pulse is applied to the primary motor cortex, TMS causes contralateral muscular responses (motor evoked potentials; MEPs). These responses can be quantified using surface electromyography and provide a measure of corticospinal excitability (see e. g. Hallett, 2007). Several studies observed that MEP amplitude was higher when emotional stimuli were presented compared with neutral control stimuli (Baumgartner, Willi, & Jäncke, 2007; Hajcak et al., 2007), suggesting a direct effect of the emotional stimuli on the motor system. Furthermore, Schutter, Hofman, and Van Honk (2008) observed that negative emotional stimuli such as faces with fearful expressions had the most pronounced effect on MEP amplitudes compared to happy faces. Similarly, van Loon, van den Wildenberg, van Stegeren, Ridderinkhof and Hajcak (2010) found that task-irrelevant negative stimuli revealed largest MEP amplitudes compared to non-emotional stimuli or positive stimuli. Taken together, these TMS studies suggest that emotions (especially negative ones) lead to a state of action readiness.

### May distinct ‘emotions’ lead to impulsivity?

So far, we have argued that impulsive actions are triggered by negative (appraised) events in general. Yet, some negative events might be more likely to trigger impulsive actions than others. In particular, it seems that impulsive actions are preferentially promoted by negative events associated with failure, frustration or regret.

Carver (2006) elaborated on the specific role of emotions on action or invigoration of behavior. According to Carver’s (2006) theoretical framework, behavior is driven by a goal and the fact that individuals aim to approach this goal while simultaneously avoid what he calls the “anti-goal” (i.e. a not-desired state). To reach the desired goal, different steps towards the goal are taken and a constant action-feedback loop compares the distance between the current state and the desired goal. Thus, individuals are continuously evaluating how well they are doing in approaching the goal and avoiding the anti-goal. This *sensed rate of progress* is compared by a ‘meta-monitoring’ loop to a criterion or standard (i.e. ‘the acceptable or desired rate of behavioral discrepancy reduction’; Carver & Scheier, 1990), which can subsequently lead to emotions: When the sensed rate of progress is the same as the criterion, no emotion arises (i.e. affect is neutral); when the sensed rate of progress is above the criterion, a positive emotion arises; and when the sensed rate of progress is below the criterion, a negative emotion arises. In this context, experiencing negative emotions becomes a cue to increase effort (to close the gap), whereas experiencing positive emotions can indicate that effort can be reduced (as the goal is (nearly) reached). Note that the action-feedback loop in Carver’s framework is similar to the comparison between current and desired state of action readiness in Frijda’s (2010) framework. However, affect arises from the meta-feedback loop (monitoring rate of progress, rather than discrepancy per se) in Carver’s framework. Furthermore, Carver (2006) argues that not every negative emotion is equivalent in terms of increased task engagement. For example, negative emotions, such as being ‘sad’, might signal that there was too much engagement and that these would lead to decreased engagement. By contrast, negative emotions such as frustration or regret might be associated with increased task engagement.

Consistent with the idea that negative emotions such as ‘frustration’ (i.e. a negative affective state that is induced by a failure to obtain an incentive or the blockage of a desired goal) or regret (i.e. the realization that another choice would have produced a better outcome, which may be a cognitive form of frustration; Reid, 1986) can invigorate behavior, a recent study observed faster responses after losses in a gambling task (Verbruggen, Chambers, Lawrence, & McLaren, 2017). In this gambling task, participants could either choose a non-gambling option (a guaranteed amount of points) or choose a gambling option (the amount was always higher than the amount associated with the non-gamble, but the probability of winning was always lower). In all experiments of the study, the initiation of the next trial was faster after a gambled loss compared to the non-gamble baseline or a gambled win.^1^ This finding was even enlarged in trials with high losses compared to low losses. Thus, it seems that losses, which were subjectively rated as negative emotional events (consistent with Wu et al., 2015), can lead to invigorated (‘impulsive’) actions as indicated by the relatively fast initiation of the subsequent trial. Similarly, Yu, Mobbs, Seymour, Rowe and Calder (2014) found that participants who did not receive an anticipated reward (i.e. the reward delivery was ‘blocked’) rated frustration levels higher and pressed the response key with more force than participants who did receive a reward (see also the seminal work on frustration by Amsel, 1958). Furthermore, these differences in Yu et al. (2014) were pronounced when the blocked reward was higher. Finally, the idea that non-reward or losses might lead to impulsive behaviors is further supported by a study of Gipson et al. (2012), investigating the link between ‘negative urgency’ (as a trait) and frustrative non-reward. They demonstrated that participants scoring high on the negative urgency scale of the UPPS questionnaire (Whiteside & Lynam, 2001) also reacted more ‘impulsively’ to unexpected non-reward (by showing an increase in operant responding) than participants scoring low on the negative urgency scale. Combined, these findings suggest that a failure to obtain a reward may invigorate behavior and lead to different kinds of impulsive acts.

Taken together, the reviewed literature suggests that impulsive actions typically arise from emotional events, and in particular from failures to obtain a reward and/or an obstruction of goal pursuit. More specifically, impulsive actions seem to arise when the individual expects reward or tries to avoid an unrewarded or punished state. This will lead to an urge to act, which is connected to psychophysiological activation (arousal). When such an urge arises, the first available action schema, a representation about the actual action that can be executed, is selected and executed even though it might be inappropriate to the situation (Evenden, 1999). This constitutes the simplest form of actions promoted by emotions, i.e., ‘impulsive actions’ (Frijda, 2010).

## Control of impulsive actions

So far, we described how negative emotionally appraised events can induce impulsive action. However, not all emotional events will lead to impulsive action. Even though some evidence suggests that negative emotions (and in particular, emotion-induced arousal) are linked to a decrease in impulse control (see below), impulses might still be overridden by alternative (‘non-impulsive’) actions or behaviors. For example, people may inhibit a prepotent response (e.g. in the coffee-machine example in **Textbox 1**, not hitting the machine when it is not working) and/or replace it with a different action (e.g. checking if the machine is plugged in). According to Frijda (2010), whether or not an impulsive action is executed, cancelled, or replaced with another action, depends on the anticipated outcome of the action and the information about the stimulus and situation (e.g. the coffee machine is always unplugged during the weekend). But how this ‘overriding process’ would work remains unclear in Frijda’s (2010) framework. This crucial control issue is addressed in the next sections.

### Distinct ways to control actions

Different ways to control (impulsive) actions have been proposed, but as mentioned above, the dominant idea in the control literature^2^ is that impulsivity is a consequence of a failure to inhibit or suppress inappropriate actions or response tendencies (Bari & Robbins, 2013; Dalley, Everitt, & Robbins, 2011; Hamilton et al., 2015; Verbruggen & Logan, 2008). However, stopping all behavioral responses is an extreme case of control (Logan, 1994), which might not always be entirely functional. For example, some TMS work indicates that response inhibition can have global effects on the motor system, even affecting task-irrelevant effectors (e.g. Badry et al., 2009). Global inhibition can also have profound after-effects, which again, may or may not be functional. Thus, some situations might require more subtle forms of control when selecting between different response options or action tendencies. More generally, questions can be raised regarding the validity of popular response-inhibition tasks (especially when neutral go stimuli and external stop signals are used; e.g. Diamond, 2013; Ridderinkhof, van den Wildenberg, & Brass, 2014), and the extent to which a lack of inhibition of prepotent responses and impulsivity should be equated (Nigg, 2017; see also Verbruggen, McLaren, & Chambers, 2014, for a critical analysis of action-control tasks). Thus, it is important to move beyond the sole response-inhibition construct in order to determine how impulsive actions are controlled in a variety of situations.

A first alternative type of impulse-control is directly connected to the origin of impulsive actions. Frijda, Ridderinkhof and Rietveld (2014) proposed that events can induce two (or more) conflicting states of action readiness (see e.g. also Cisek & Kalaska, 2010, who also argued that multiple response options or action representations are activated before a single one is selected and executed). Such competing states of action readiness may cancel each other out, resulting in reduced or absent overt actions. This kind of control is considered to be an inherent property to the action system, and does not involve extra ‘top-down’ processes. Note though that these action-readiness conflicts may still lead to impulsive behavior, when the competition does not succeed in ‘dampening’ of the action tendencies (Frijda et al., 2014) and the first available action schema becomes selected.

Another possible way to control actions involves biasing this action-tendency conflict or competition within a trial (Frijda et al., 2014). This may involve biasing task-relevant information (e.g. via task units or goals; Cohen, Dunbar, & McClelland, 1990) and suppression of task-irrelevant information or responses. The latter idea is supported by distributional analyses of performance data in tasks such as the Simon task (in which participants respond with left/right responses to a feature of stimulus that is presented on the left or right of the screen). For example, Ridderinkhof (2002) found that response-compatibility or interference effects were smaller for slower responses (and could even reverse; i.e. ‘faster’ responses on incongruent trials compared with congruent trials). He attributed this pattern to the selective suppression of response activation resulting from task-irrelevant information (i.e. the location in a Simon task). However, this selective suppression takes some time to build up (hence the decrease over time).

The different control mechanisms are not mutually exclusive, and conflict might be resolved in different (complementary) ways. Indeed, Wiecki and Frank (2013) proposed that three pathways are involved when executing a response in a conflict situation. There is a *striatal Go pathway*, which selectively facilitates a response, *a striatal NoGo pathway*, which suppresses prepotent responses, and *the hyperdirect pathway*, which suppresses globally all responses and regulates the gating threshold of a response. This hyperdirect pathway is activated when conflict is detected. When all possible responses are suppressed, and thus slowed down, the conflict can be solved and the correct response can be selected. Thus, in Wiecki and Frank’s model there can be both a suppression of a prepotent response and a general suppression of all responses to resolve conflict within a trial.

So far, we only discussed within-trial adjustments. But adjustments can also be made across trials. For example, many psychological theories (Botvinick, Braver, Barch, Carter, & Cohen, 2001; Laming, 1979; Rabbitt & Rodgers, 1977) assume that a cognitive control system further alters or biases the settings of lower-level systems when people make an error, when conflict is detected, or more generally, when outcomes are suboptimal. For example, people often slow down after they make an error (‘post-error slowing’). Such slowing has been observed in many cognitive tasks (including simple two choice reaction time tasks, conflict tasks such as the Flanker and Simon tasks, or response-inhibition tasks such as the stop-signal task), and is usually attributed to performance-related adjustments in task-relevant processing pathways. Such adjustments usually increase response latencies (i.e. people become more cautious) and may reduce the likelihood of further errors or further improve task performance in subsequent trials (Dutilh et al., 2012, for further reading on between trial adaptation see also Duthoo, Abrahamse, Braem, Boehler, & Notebaert, 2014).

### Emotional foundations of impulse control

Recent evidence suggests that emotional states might also constitute an integral part of the cognitive-control and performance-adjustments processes (Dreisbach & Fischer, 2012; Fritz & Dreisbach, 2013; Riesel, Weinberg, Endrass, Kathmann, & Hajcak, 2012; van Steenbergen, Band, & Hommel, 2009). In other words, emotions might play a pivotal role not only in the origin but also in the control of (impulsive) action.

Several studies have focused on the causal role of emotions in response-inhibition tasks or other related impulse-control paradigms (e. g. Rebetez, Rochat, Billieux, Gay, & Van der Linden, 2015; Schulz et al., 2007; Verbruggen & De Houwer, 2007). These studies indicate that emotional arousal in particular can have a major impact on response inhibition (i.e. the first control type described in the previous section). For example, Verbruggen and de Houwer (2007) used a stop-signal task and presented emotional pictures before the actual task stimuli. In a first experiment, they found that both positive and negative emotional pictures impaired go and stop performance (i.e. both go and stop latencies were prolonged when emotional pictures were presented, compared with neutral images). A second experiment showed that high-arousal pictures impaired the performance more than low-arousal pictures. Valence had only little effect in these experiments. This led Verbruggen and De Houwer (2007) to conclude that emotionally arousing pictures are distracting, generally impairing performance in the stop-signal task. Similarly, De Houwer and Tibboel (2010), found impaired performance in a go/no-go task (another popular response-inhibition task) after arousing pictures. They also did not find a significant effect of valence (although their data showed that the effect of arousal was numerically stronger for negative compared to positive pictures). Pessoa (2009) suggested that emotions might influence executive control processes in two different ways corresponding to either ‘perceptual’ or ‘executive’ processing. First, emotional content enhances perceptual processing of stimuli. This would lead to task improvements when low-threat emotional stimuli are task relevant (e.g. Pessoa, Padmala, Kenzer, & Bauer, 2012), but impairments when these are task-irrelevant. Second, Pessoa (2009) proposes that high-treat or high-arousal stimuli will also require ‘attentional’ or ‘effortful’ control. As executive control capacity is assumed to be limited in Pessoa’s framework, such ‘executive’ processing of emotional (threatening) stimuli would impair executive or cognitive processes, such as response inhibition.

There is evidence that information biasing or adjustments of task settings are also influenced by emotional stimuli. Van Steenbergen et al. (2009) presented random monetary gains or losses as feedback in a Flanker task, and found that the random gains after a conflict trial (leading to a positive mood) reduced subsequent conflict adaptation. By contrast, negative emotion seemed to increase conflict adaptation (Dreisbach & Fischer, 2012) and post-error slowing was enlarged when errors were punished, leading to a more negative mood state (Riesel et al., 2012). Furthermore, Fritz & Dreisbach (2013) found that neutral stimuli following an incongruent Stroop trial (in which target and distractor required different responses) were judged as more negative than neutral stimuli following a non-conflict trial (in which target and distractor required the same response). Finally, psychophysiological studies using facial EMG found that the degree of corrugator supercilii activation (see above) in response to an error correlated positively with post-error slowing (Lindström et al., 2013; Saunders & Inzlicht, 2015). Based on these and other related findings (e.g. Braem et al., 2017), it has been argued that conflict and errors elicit negative emotion, which acts as a trigger for subsequent cognitive control adjustments.

### Towards an integration

Conflict and performance-adjustment studies have shown that the experience of negative emotions or the confrontation with negatively valenced stimuli can increase cognitive control (or potentially promote a proactive mode of information processing) under at least some circumstances. In the framework of Carver (2006) and Frijda (2010), this might be functional because increased control can help to narrow the gap between the current state and the desired state. But as discussed in the previous sections, there is also a corpus of evidence suggesting that negative (emotional) events can promote impulsive behavior (Dyson, Sundvall, Forder, & Douglas, 2018; Verbruggen et al., 2017; Yu et al., 2014). Saunders and Inzlicht (2015) proposed the *shifting priorities model* to explain how a negative event such as an error or failure may result in distinct after-effects. According to these authors, a desire for gratification will emerge and the individual will try to return to ‘cognitive comfort’ (i.e. a state which is characterized by an acceptable (and thus low) level of negative emotion) when they experience a prolonged period of unrewarded control processes. Thus, Saunders and Inzlicht’s model also starts from a discrepancy between the current state (e.g. having no coffee) and a desired state (e.g. drinking coffee). Importantly, however, the best strategy to re-gain cognitive comfort would differ according to contextual and environmental factors. For example, after an error in a controllable decision task, individuals might increase cognitive control in an attempt to achieve cognitive comfort. In contrast, when individuals have no control, they might experience fatigue and hence, generally decrease cognitive control. This can result in less effort (or even no behavior) when the individuals perceive the problem as exceeding their own abilities or as unsolvable (Gendolla, Abele, & Krüsken, 2001; Mikulincer, 1988). Uncontrollable situations may even lead to more ‘impulsive’ behavior (as we have seen above). Thus, Saunders and Inzlicht (2015) suggested that vigor or fatigue at a control level are dependent on the task context.

The ‘controllability’ idea recently received support from Dyson et al. (2018). Using a rock, paper, scissor game, these authors tested whether predictability (i.e. can strategies be applied or not?) influenced impulsive actions after negative outcomes. In the standard unpredictable condition (in which participants could not derive the strategy used by the other player), Dyson et al. (2018) observed shorter response latencies after a loss than after a win. This is consistent with the findings of Verbruggen et al. (2017). Importantly, the reversed effect (i.e. longer latencies after a loss than after a win) was observed when the opponent’s strategy was predictable and participants could apply a strategy themselves. Similarly, this can be observed with errors on trial-by-trial basis. Damaso, Williams and Heathcote (in press) found that the type of error determined whether participants slowed down or not after an error. When errors resulted from responding too quickly (favoring speed over accuracy), post-error slowing was observed. However, when the errors resulted from poor stimulus quality, no post-error slowing was observed. Indeed, it could be argued that there is no point in slowing down when the evidence quality remains the same over time. Thus, these findings suggest that slowing depends on the task characteristics.

Sone findings even suggest that general beliefs can influence control adjustments. For example, Rigoni, Wilquin, Brass and Burle (2013) identified an influence of perceived control on post-error slowing. In this between-subject study, they manipulated the belief in free will, after which participants performed a Simon task. The authors found reduced post-error slowing in the group with reduced belief in free will. Furthermore, a follow-up study showed that such beliefs modulated ERPs typically associated with error monitoring (Rigoni, Pourtois, & Brass, 2015). Thus, it seems that the response to a negative event such as an error or a loss might be modulated by the perceived control of the participants.

## Conclusions

As reflected in recent reviews, most clinical, cognitive, neuroscience, and animal studies on impulsive action have examined how humans and non-human animals can stop or withhold a prepotent action in response to an external signal (i.e. response inhibition). This research has led researchers to propose that response inhibition may serve as an endophenotype for impulsive action, arguing that variation in the effectiveness of inhibitory control underlie impulsive behaviors in the real world. However, the hitherto strong focus on externally-triggered response inhibition has resulted in narrow views of impulse control.

**Figure 1:**
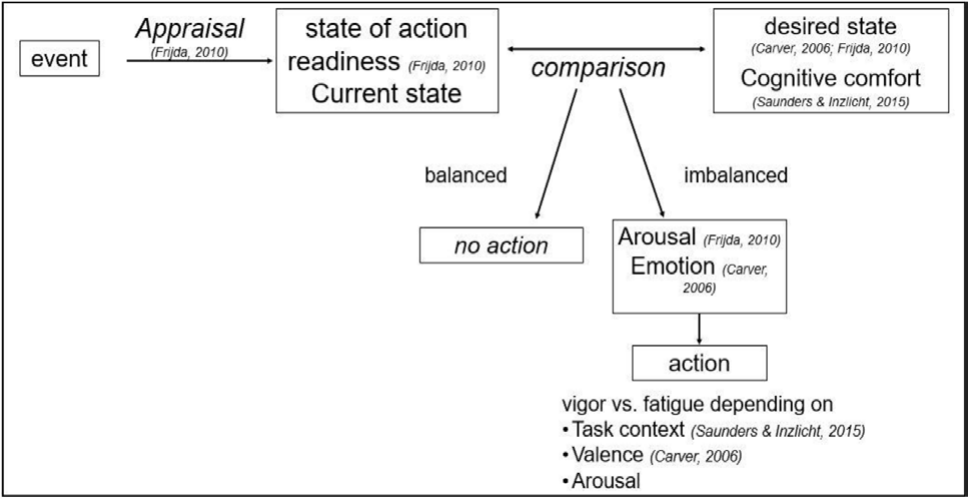
Summary of the main theoretical accounts of impulsive action. All theories assume that the comparison between the current state and the desired state (e.g. cognitive comfort) leads to actions. However, the theories assume different reasons for an action being invigorated or not.

In this critical review, we elaborated on how impulsive actions originate and how these can be controlled in different ways. We focused in particular on the role of negative events. We discussed how events are constantly appraised, and how discrepancies between current and desired goals or states can induce action tendencies or changes in response strategies and control settings. In this view, impulsive actions are ‘emotional’ actions. We also described different control processes that can be engaged in different situations: global suppression of all actions (as studies in traditional response-inhibition tasks), direct competition between different states of action readiness, and biasing of task-relevant information. There is evidence that these control processes can also be influenced by distinct emotions or moods. Whether or not emotion would enhance or impair impulse control might depend on the task or context (e.g. controllability of the task) and whether or not the emotional events are task-relevant or not. Thus, as summarized in Figure 1, both the origin and the control of impulsive actions seem to be closely linked to negative (emotional) events, in particular the difference between a current state and a desired state, which in turn depends also on other circumstances such as the perceived control. Further outstanding theoretical questions are highlighted in **Textbox 2**.

**Textbox 2: Key outstanding theoretical questions.**

Research on traitimpulsivity suggests that negative emotions can lead to impulsive actions in everyday life. However, this critical review indicates there are still several open questions. Here we highlight three questions that deserve particular attention.

**What is the emotional response to suboptimal behavioral outcomes, and what role doemotional arousal play?**

Negative emotional events have been associated with impulsivity andrestraint. According to some authors (see main text),distinct negative emotional eventsmight lead to distinct outcomes. Arousal may also play a critical role. It is therefore essential to study the nature of the ‘emotional’ responseand the behaviorafter non-reward to understand how actions become invigorated.

**How do internal beliefs influence the emotional response after a negative outcome?**

Anevent becomes ‘emotional’as soon as it is appraised by the individual. Nevertheless, the emotionaland behavioralresponse might depend on whether the participants feel responsible for the outcome or not (Koban & Pourtois, 2014), even though the event itself remains the same. Frijda (2010) also argued that the process of appraisal might be influenced by individual experiences and different beliefs. This link between internal beliefs and the origin and control of impulsive action still has to be explored though.

**What are the mechanisms underlying the effect of post-loss speeding?**

Failures to obtain a reward can induce frustration and invigorate subsequent behavior. However, in previous research, ‘frustration’ referred both to the effect (i.e. increased response vigor or impulsivity) and the cause, and a mechanistic account is missing. Therefore, future research is required to explore (at a mechanistic level) how non-reward induces changes in the motor system.

## Footnotes

Note, that we recently were able to replicate these results in a short pilot experiment. Raw data can be found on OSF (osf.io/7xbth).

For a discussion of the (sometimes subtle) differences between executive control, self-control and similar concepts, see Nigg (2017).

## References

[1] Amsel, A. (1958). The role of frustrative nonreward in noncontinuous reward situations. Psychological Bulletin, 55(2), 102–119. DOI: 10.1037/h0043125 13527595

[2] Badry, R., Mima, T., Aso, T., Nakatsuka, M., Abe, M., Fathi, D., … Fukuyama, H. (2009). Suppression of human cortico-motoneuronal excitability during the Stop-signal task. Clinical Neurophysiology, 120(9), 1717–1723. DOI: 10.1016/j.clinph.2009.06.027 19683959

[3] Bari, A., & Robbins, T. W. (2013). Inhibition and impulsivity: Behavioral and neural basis of response control. Progress in Neurobiology, 108, 44–79. DOI: 10.1016/j.pneurobio.2013.06.005 23856628

[4] Baumgartner, T., Willi, M., & Jäncke, L. (2007). Modulation of corticospinal activity by strong emotions evoked by pictures and classical music: A transcranial magnetic stimulation study. NeuroReport, 18(3), 261–265. DOI: 10.1097/WNR.0b013e328012272e 17314668

[5] Berg, J. M., Latzman, R. D., Bliwise, N. G., & Lilienfeld, S. O. (2015). Parsing the heterogeneity of impulsivity: A meta-analytic review of the behavioral implications of the UPPS for psychopathology. Psychological Assessment, 27(4), 1129–1146. DOI: 10.1037/pas0000111 25822833

[6] Bezdjian, S., Baker, L. A., & Tuvblad, C. (2011). Genetic and environmental influences on impulsivity: A meta-analysis of twin, family and adoption studies. Clinical Psychology Review, 31(7), 1209–1223. DOI: 10.1016/j.cpr.2011.07.005 21889436 PMC3176916

[7] Billieux, J., Gay, P., Rochat, L., & Van der Linden, M. (2010). The role of urgency and its underlying psychological mechanisms in problematic behaviours. Behaviour Research and Therapy, 48(11), 1085–1096. DOI: 10.1016/j.brat.2010.07.008 20708729

[8] Botvinick, M. M., Braver, T. S., Barch, D. M., Carter, C. S., & Cohen, J. D. (2001). Conflict monitoring and cognitive control. Psychological Review, 108(3), 624–652. DOI: 10.1037/0033-295X.108.3.624 11488380

[9] Braem, S., King, J. A., Korb, F. M., Krebs, R. M., Notebaert, W., & Egner, T. (2017). The Role of Anterior Cingulate Cortex in the Affective Evaluation of Conflict. Journal of Cognitive Neuroscience, 29(1), 137–149. DOI: 10.1162/jocn_a_01023 27575278 PMC5341786

[10] Carver, C. S. (2006). Approach, Avoidance, and the Self-Regulation of Affect and Action. Motivation and Emotion, 30(2), 105–110. DOI: 10.1007/s11031-006-9044-7

[11] Carver, C. S., & Scheier, M. F. (1990). Origins and functions of positive and negative affect: A control-process view. Psychological Review, 97(1), 19–35. DOI: 10.1037/0033-295X.97.1.19

[12] Cisek, P., & Kalaska, J. F. (2010). Neural Mechanisms for Interacting with a World Full of Action Choices. Annual Review of Neuroscience, 33(1), 269–298. DOI: 10.1146/annurev.neuro.051508.135409 20345247

[13] Cohen, J. D., Dunbar, K., & McClelland, J. L. (1990). On the control of automatic processes: A parallel distributed processing account of the Stroop effect. Psychological Review, 97(3), 332 DOI: 10.1037/0033-295X.97.3.332 2200075

[14] Costa, P. T., Jr, & McCrae, R. R. (1992). Revised NEO personality inventory (NEO-PI-R) and NEO five-factor (NEO-FFI) inventory professional manual Odessa, Fl: PAR.

[15] Cyders, M. A., & Coskunpinar, A. (2012). The relationship between self-report and lab task conceptualizations of impulsivity. Journal of Research in Personality, 46(1), 121–124. DOI: 10.1016/j.jrp.2011.11.005

[16] Cyders, M. A., & Smith, G. T. (2007). Mood-based rash action and its components: Positive and negative urgency. Personality and Individual Differences, 43(4), 839–850. DOI: 10.1016/j.paid.2007.02.008

[17] Cyders, M. A., & Smith, G. T. (2008). Emotion-based dispositions to rash action: Positive and negative urgency. Psychological Bulletin, 134(6), 807–828. DOI: 10.1037/a0013341 18954158 PMC2705930

[18] Dalley, J. W., Everitt, B. J., & Robbins, T. W. (2011). Impulsivity, Compulsivity, and Top-Down Cognitive Control. Neuron, 69(4), 680–694. DOI: 10.1016/j.neuron.2011.01.020 21338879

[19] Dalley, J. W., & Robbins, T. W. (2017). Fractionating impulsivity: Neuropsychiatric implications. Nature Reviews Neuroscience, 18(3), 158–171. DOI: 10.1038/nrn.2017.8 28209979

[20] Damaso, K., Williams, P. & Heathcote, A. (in press). Evidence for different types of errors being associated with different types of post-error changes. Psychonomic Bulletin & Review. 10.3758/s13423-019-01675-w 31907850

[21] De Houwer, J., & Tibboel, H. (2010). Stop what you are not doing! Emotional pictures interfere with the task not to respond. Psychonomic Bulletin & Review, 17(5), 699–703. DOI: 10.3758/PBR.17.5.699 21037169

[22] Diamond, A. (2013). Executive Functions. Annual Review of Psychology, 64(1), 135–168. DOI: 10.1146/annurev-psych-113011-143750 PMC4084861 23020641

[23] Donders, F. C. (1969). On the speed of mental processes. Acta Psychologica, 30, 412–431. DOI: 10.1016/0001-6918(69)90065-1 5811531

[24] Dreisbach, G., & Fischer, R. (2012). The role of affect and reward in the conflict-triggered adjustment of cognitive control. Frontiers in Human Neuroscience, 6 DOI: 10.3389/fnhum.2012.00342 PMC3533233 23293597

[25] Duthoo, W., Abrahamse, E. L., Braem, S., Boehler, C. N., & Notebaert, W. (2014). The heterogeneous world of congruency sequence effects: An update. Frontiers in Psychology, 5 DOI: 10.3389/fpsyg.2014.01001 PMC4158803 25250005

[26] Dutilh, G., Vandekerckhove, J., Forstmann, B. U., Keuleers, E., Brysbaert, M., & Wagenmakers, E.-J. (2012). Testing theories of post-error slowing. Attention, Perception, & Psychophysics, 74(2), 454–465. DOI: 10.3758/s13414-011-0243-2 PMC3283767 22105857

[27] Dyson, B. J., Sundvall, J., Forder, L., & Douglas, S. (2018). Failure generates impulsivity only when outcomes cannot be controlled. Journal of Experimental Psychology: Human Perception and Performance. DOI: 10.1037/xhp0000557 30024223

[28] Elkins-Brown, N., Saunders, B., & Inzlicht, M. (2016). Error-related electromyographic activity over the corrugator supercilii is associated with neural performance monitoring: Facial EMG relates to PE. Psychophysiology, 53(2), 159–170. DOI: 10.1111/psyp.12556 26470645

[29] Evenden, J. L. (1999). Varieties of impulsivity. Psychopharmacology, 146(4), 348–361. DOI: 10.1007/PL00005481 10550486

[30] Frijda, N. H. (2010). Impulsive action and motivation. Biological Psychology, 84(3), 570–579. DOI: 10.1016/j.biopsycho.2010.01.005 20064583

[31] Frijda, N. H., Ridderinkhof, K. R., & Rietveld, E. (2014). Impulsive action: Emotional impulses and their control. Frontiers in Psychology, 5 DOI: 10.3389/fpsyg.2014.00518 PMC4040919 24917835

[32] Fritz, J., & Dreisbach, G. (2013). Conflicts as aversive signals: Conflict priming increases negative judgments for neutral stimuli. Cognitive, Affective, & Behavioral Neuroscience, 13(2), 311–317. DOI: 10.3758/s13415-012-0147-1 23307475

[33] Gay, P., Rochat, L., Billieux, J., d’Acremont, M., & Van der Linden, M. (2008). Heterogeneous inhibition processes involved in different facets of self-reported impulsivity: Evidence from a community sample. Acta Psychologica, 129(3), 332–339. DOI: 10.1016/j.actpsy.2008.08.010 18851842

[34] Gendolla, G. H. E., Abele, A. E., & Krüsken, J. (2001). The informational impact of mood on effort mobilization: A study of cardiovascular and electrodermal responses. Emotion, 1(1), 12–24. DOI: 10.1037//1528-3542.1.1.12 12894808

[35] Gipson, C. D., Beckmann, J. S., Adams, Z. W., Marusich, J. A., Nesland, T. O., Yates, J. R., … Bardo, M. T. (2012). A translational behavioral model of mood-based impulsivity: Implications for substance abuse. Drug and Alcohol Dependence, 122(1–2), 93–99. DOI: 10.1016/j.drugalcdep.2011.09.014 21975194 PMC3270200

[36] Hajcak, G., Molnar, C., George, M. S., Bolger, K., Koola, J., & Nahas, Z. (2007). Emotion facilitates action: A transcranial magnetic stimulation study of motor cortex excitability during picture viewing. Psychophysiology, 44(1), 91–97. DOI: 10.1111/j.1469-8986.2006.00487.x 17241144

[37] Hallett, M. (2007). Transcranial Magnetic Stimulation: A Primer. Neuron, 55(2), 187–199. DOI: 10.1016/j.neuron.2007.06.026 17640522

[38] Hamilton, K. R., Littlefield, A. K., Anastasio, N. C., Cunningham, K. A., Fink, L. H. L., Wing, V. C., … Potenza, M. N. (2015). Rapid-response impulsivity: Definitions, measurement issues, and clinical implications. Personality Disorders: Theory, Research, and Treatment, 6(2), 168–181. DOI: 10.1037/per0000100 PMC4476624 25867840

[39] Hofmann, W., Friese, M., & Strack, F. (2009). Impulse and Self-Control From a Dual-Systems Perspective. Perspectives on Psychological Science, 4(2), 162–176. DOI: 10.1111/j.1745-6924.2009.01116.x 26158943

[40] Inzlicht, M., Bartholow, B. D., & Hirsh, J. B. (2015). Emotional foundations of cognitive control. Trends in Cognitive Sciences, 19(3), 126–132. DOI: 10.1016/j.tics.2015.01.004 25659515 PMC4348332

[41] Kirby, K. N., & Maraković, N. N. (1996). Delay-discounting probabilistic rewards: Rates decrease as amounts increase. Psychonomic Bulletin & Review, 3(1), 100–104. DOI: 10.3758/BF03210748 24214810

[42] Koban, L., & Pourtois, G. (2014). Brain systems underlying the affective and social monitoring of actions: An integrative review. Neuroscience & Biobehavioral Reviews, 46, 71–84. DOI: 10.1016/j.neubiorev.2014.02.014 24681006

[43] Laming, D. (1979). Choice reaction performance following an error. Acta Psychologica, 43(3), 199–224. DOI: 10.1016/0001-6918(79)90026-X 495175

[44] Lindström, B. R., Mattsson-Mårn, I. B., Golkar, A., & Olsson, A. (2013). In Your Face: Risk of Punishment Enhances Cognitive Control and Error-Related Activity in the Corrugator Supercilii Muscle. PLoS ONE, 8(6), e65692 DOI: 10.1371/journal.pone.0065692 23840356 PMC3694071

[45] Logan, G. D. (1994). On the ability to inhibit thought and action: A users’ guide to the stop signal paradigm In D. Dagenbach & T. H. Carr (Eds.), Inhibitory processes in attention, memory, and language (pp. 189–239). San Diego, CA: Academic Press.

[46] Logan, G. D., & Cowan, W. B. (1984). On the Ability to Inhibit Thought and Action: A Theory of an Act of Control. Psychological Review, 91(3), 295–327. DOI: 10.1037/0033-295X.91.3.295 24490789

[47] Logan, G. D., Van Zandt, T., Verbruggen, F., & Wagenmakers, E.-J. (2014). On the ability to inhibit thought and action: General and special theories of an act of control. Psychological Review, 121(1), 66–95. DOI: 10.1037/a0035230 24490789

[48] Mauss, I. B., & Robinson, M. D. (2009). Measures of emotion: A review. Cognition & Emotion, 23(2), 209–237. DOI: 10.1080/02699930802204677 19809584 PMC2756702

[49] Metcalfe, J., & Mischel, W. (1999). A hot/cool-system analysis of delay of gratification: Dynamics of willpower. Psychological Review, 106(1), 3–19. DOI: 10.1037/0033-295X.106.1.3 10197361

[50] Mikulincer, M. (1988). Reactance and helplessness following exposure to unsolvable problems: The effects of attributional style. Journal of Personality and Social Psychology, 54(4), 679–686. DOI: 10.1037//0022-3514.54.4.679 3367284

[51] Nigg, J. T. (2017). Annual Research Review: On the relations among self-regulation, self-control, executive functioning, effortful control, cognitive control, impulsivity, risk-taking, and inhibition for developmental psychopathology. Journal of Child Psychology and Psychiatry, 58(4), 361–383. DOI: 10.1111/jcpp.12675 28035675 PMC5367959

[52] Pessoa, L. (2009). How do emotion and motivation direct executive control? Trends in Cognitive Sciences, 13(4), 160–166. DOI: 10.1016/j.tics.2009.01.006 19285913 PMC2773442

[53] Pessoa, L., Padmala, S., Kenzer, A., & Bauer, A. (2012). Interactions between cognition and emotion during response inhibition. Emotion, 12(1), 192–197. DOI: 10.1037/a0024109 21787074 PMC3208031

[54] Poldrack, R. (2006). Can cognitive processes be inferred from neuroimaging data? Trends in Cognitive Sciences, 10(2), 59–63. DOI: 10.1016/j.tics.2005.12.004 16406760

[55] Rabbitt, P., & Rodgers, B. (1977). What does a Man do after he Makes an Error? An Analysis of Response Programming. Quarterly Journal of Experimental Psychology, 29(4), 727–743. DOI: 10.1080/14640747708400645

[56] Rebetez, M. M. L., Rochat, L., Billieux, J., Gay, P., & Van der Linden, M. (2015). Do emotional stimuli interfere with two distinct components of inhibition? Cognition and Emotion, 29(3), 559–567. DOI: 10.1080/02699931.2014.922054 24885111

[57] Reid, R. L. (1986). The psychology of the near miss. Journal of Gambling Behavior, 2(1), 32–39. DOI: 10.1007/BF01019932

[58] Ridderinkhof, K. R. (2002). Micro- and macro-adjustments of task set: Activation and suppression in conflict tasks. Psychological Research, 66(4), 312–323. DOI: 10.1007/s00426-002-0104-7 12466928

[59] Ridderinkhof, K. R., van den Wildenberg, W. P. M., & Brass, M. (2014). “Don’t” versus “Won’t”: Principles, mechanisms, and intention in action inhibition. Neuropsychologia, 65, 255–262. DOI: 10.1016/j.neuropsychologia.2014.09.005 25218168

[60] Riesel, A., Weinberg, A., Endrass, T., Kathmann, N., & Hajcak, G. (2012). Punishment has a lasting impact on error-related brain activity: Punishment modulates error monitoring. Psychophysiology, 49(2), 239–247. DOI: 10.1111/j.1469-8986.2011.01298.x 22092041

[61] Rigoni, D., Pourtois, G., & Brass, M. (2015). ‘Why should I care?’ Challenging free will attenuates neural reaction to errors. Social Cognitive and Affective Neuroscience, 10(2), 262–268. DOI: 10.1093/scan/nsu068 24795441 PMC4321631

[62] Rigoni, D., Wilquin, H., Brass, M., & Burle, B. (2013). When errors do not matter: Weakening belief in intentional control impairs cognitive reaction to errors. Cognition, 127(2), 264–269. DOI: 10.1016/j.cognition.2013.01.009 23466640

[63] Russell, J. A., & Barrett, L. F. (1999). Core Affect, Prototypical Emotional Episodes, and Other Things Called Emotion: Dissecting the Elephant. Journal of Personality and Social Psychology, 76(5), 805–819. DOI: 10.1037/0022-3514.76.5.805 10353204

[64] Saunders, B., & Inzlicht, M. (2015). How Variation in Affect Underlies Effective Self-Control In T. Braver (Ed.), Motivation and cognitive control. New York, NY: Taylor & Francis/Routledge.

[65] Schulz, K., Fan, J., Magidina, O., Marks, D., Hahn, B., & Halperin, J. (2007). Does the emotional go/no-go task really measure behavioral inhibition? Convergence with measures on a non-emotional analog. Archives of Clinical Neuropsychology, 22(2), 151–160. DOI: 10.1016/j.acn.2006.12.001 17207962 PMC2562664

[66] Schutter, D. J. L. G., Hofman, D., & Van Honk, J. (2008). Fearful faces selectively increase corticospinal motor tract excitability: A transcranial magnetic stimulation study. Psychophysiology, 45(3), 345–348. DOI: 10.1111/j.1469-8986.2007.00635.x 18221448

[67] Selby, E. A., Anestis, M. D., & Joiner, T. E. (2008). Understanding the relationship between emotional and behavioral dysregulation: Emotional cascades. Behaviour Research and Therapy, 46(5), 593–611. DOI: 10.1016/j.brat.2008.02.002 18353278

[68] Simpson, A., Riggs, K. J., Beck, S. R., Gorniak, S. L., Wu, Y., Abbott, D., & Diamond, A. (2012). Refining the understanding of inhibitory processes: How response prepotency is created and overcome: How response prepotency is created and overcome. Developmental Science, 15(1), 62–73. DOI: 10.1111/j.1467-7687.2011.01105.x 22251293 PMC3405835

[69] Smith, G. T., & Cyders, M. A. (2016). Integrating affect and impulsivity: The role of positive and negative urgency in substance use risk. Drug and Alcohol Dependence, 163, S3–S12. DOI: 10.1016/j.drugalcdep.2015.08.038 27306729 PMC4911536

[70] van Gaalen, M. M., Brueggeman, R. J., Bronius, P. F. C., Schoffelmeer, A. N. M., & Vanderschuren, L. J. M. J. (2006). Behavioral disinhibition requires dopamine receptor activation. Psychopharmacology, 187(1), 73–85. DOI: 10.1007/s00213-006-0396-1 16767417

[71] van Gaalen, M. M., van Koten, R., Schoffelmeer, A. N. M., & Vanderschuren, L. J. M. J. (2006). Critical Involvement of Dopaminergic Neurotransmission in Impulsive Decision Making. Biological Psychiatry, 60(1), 66–73. DOI: 10.1016/j.biopsych.2005.06.005 16125144

[72] van Loon, A. M., van den Wildenberg, W. P. M., van Stegeren, A. H., Ridderinkhof, K. R., & Hajcak, G. (2010). Emotional stimuli modulate readiness for action: A transcranial magnetic stimulation study. Cognitive, Affective, & Behavioral Neuroscience, 10(2), 174–181. DOI: 10.3758/CABN.10.2.174 20498342

[73] van Steenbergen, H., Band, G. P. H., & Hommel, B. (2009). Reward Counteracts Conflict Adaptation: Evidence for a Role of Affect in Executive Control. Psychological Science, 20(12), 1473–1477. DOI: 10.1111/j.1467-9280.2009.02470.x 19906127

[74] Verbruggen, F., Aron, A. R., Band, G. P., Beste, C., Bissett, P. G., Brockett, A. T., … Boehler, C. N. (2019). A consensus guide to capturing the ability to inhibit actions and impulsive behaviors in the stop-signal task. ELife, 8, e46323 DOI: 10.7554/eLife.46323 31033438 PMC6533084

[75] Verbruggen, F., Chambers, C. D., Lawrence, N. S., & McLaren, I. P. L. (2017). Winning and losing: Effects on impulsive action. Journal of Experimental Psychology: Human Perception and Performance, 43(1), 147 DOI: 10.1037/xhp0000284 27808548 PMC5178881

[76] Verbruggen, F., Chambers, C. D., & Logan, G. D. (2013). Fictitious Inhibitory Differences: How Skewness and Slowing Distort the Estimation of Stopping Latencies. Psychological Science, 24(3), 352–362. DOI: 10.1177/0956797612457390 23399493 PMC3724271

[77] Verbruggen, F., & De Houwer, J. (2007). Do emotional stimuli interfere with response inhibition? Evidence from the stop signal paradigm. Cognition & Emotion, 21(2), 391–403. DOI: 10.1080/02699930600625081

[78] Verbruggen, F., & Logan, G. D. (2008). Response inhibition in the stop-signal paradigm. Trends in Cognitive Sciences, 12(11), 418–424. DOI: 10.1016/j.tics.2008.07.005 18799345 PMC2709177

[79] Verbruggen, F., McLaren, I. P. L., & Chambers, C. D. (2014). Banishing the Control Homunculi in Studies of Action Control and Behavior Change. Perspectives on Psychological Science, 9(5), 497–524. DOI: 10.1177/1745691614526414 25419227 PMC4232338

[80] Verbruggen, F., McLaren, R., Pereg, M., & Meiran, N. (2018). Structure and Implementation of Novel Task Rules: A Cross-Sectional Developmental Study. Psychological Science, 29(7), 1113–1125. DOI: 10.1177/0956797618755322 29746205 PMC6247441

[81] Whiteside, S. P., & Lynam, D. R. (2001). The Five Factor Model and impulsivity: Using a structural model of personality to understand impulsivity. Personality and Individual Differences, 30(4), 669–689. DOI: 10.1016/S0191-8869(00)00064-7

[82] Wiecki, T. V., & Frank, M. J. (2013). A computational model of inhibitory control in frontal cortex and basal ganglia. Psychological Review, 120(2), 329–355. DOI: 10.1037/a0031542 23586447

[83] Wilbertz, T., Deserno, L., Horstmann, A., Neumann, J., Villringer, A., Heinze, H.-J., … Schlagenhauf, F. (2014). Response inhibition and its relation to multidimensional impulsivity. NeuroImage, 103, 241–248. DOI: 10.1016/j.neuroimage.2014.09.021 25241087

[84] Wu, Y., van Dijk, E., & Clark, L. (2015). Near-wins and near-losses in gambling: A behavioral and facial EMG study: Near-wins and near-losses. Psychophysiology, 52(3), 359–366. DOI: 10.1111/psyp.12336 25234840 PMC4510820

[85] Yu, R., Mobbs, D., Seymour, B., Rowe, J. B., & Calder, A. J. (2014). The neural signature of escalating frustration in humans. Cortex, 54, 165–178. DOI: 10.1016/j.cortex.2014.02.013 24699035

